# Grassroots innovation and social innovation in perspective

**DOI:** 10.3389/fsoc.2023.1247293

**Published:** 2023-10-27

**Authors:** Karina Maldonado-Mariscal

**Affiliations:** Department of Social Sciences, Social Research Center (sfs), TU Dortmund University, Dortmund, Germany

**Keywords:** grassroots innovation, social innovation, review, critical perspectives, innovation

## Abstract

This article provides a comprehensive review of social innovation and grassroots innovation over the last 5 years, offering a detailed analysis of both concepts. This study explores the integration of grassroots innovation and social innovation based on an extensive literature review. It examines five dimensions within the literature: key fields, disciplines, actors, geographical areas and theoretical frameworks. Despite significant research in recent decades, there is a notable gap of literature devoted to grassroots innovation and its position within discourse of social innovation. This paper explores the differences and similarities between the concepts of social innovation and grassroots innovation in order to better understand the use of both concepts, the cases in which they are used and possible complementarities. The main findings of the literature on combining the concepts of social innovation and grassroots innovation focus on social enterprises, while research on grassroots innovation as a stand-alone concept focuses on community-led initiatives, civil society organisations, cooperatives and local leaders. Geographically, India plays a very important role in grassroots and social innovation research, followed by Brazil and Spain. In terms of theoretical approach, the combination of social innovation and grassroots innovation has a strong sociological focus, emphasising theories of social practice, collective action, solidarity and community. In contrast, the theoretical frameworks of grassroots innovation are more anchored in power relations and socio-technical transitions, including, for example, resistance to innovation. Grassroots innovation offers practical insights into understanding innovation through the lenses of grassroots and community-based social change. Similarly, social innovation can contribute to the debate on grassroots innovations by understanding not only the agency of actors, but also the innovation ecosystem, actors and types of innovation. Further empirical research on the understanding and application of both concepts in the global North and South in academic discourse offers great potential, therefore potential research questions have been raised here for further investigation.

## 1. Introduction

Current research on innovation underlines the importance of adopting a critical perspective that avoids a technology-focused understanding of innovation as well as a positive impact in all cases. For example, over the past decade, scholars have highlighted a prevalent “pro-innovation bias” (Godin and Vinck, 2017) which tends to portray innovation as inherently positive. Other researchers acknowledge both the positive and negative aspects of innovation (Dziurski, 2021). This article takes a reflective approach to explore the relationship between social innovation and grassroots innovation, focusing specifically on grassroots innovation. This paper is important to better understand grassroots innovation and to better define the boundaries of social innovation. By recognizing and explaining the connections between these two concepts, the article sheds light on their significance. Social innovation represented a new innovation paradigm in the 2010s (Maldonado-Mariscal and Alijew, 2023), shifting from a market-driven perspective to a social-oriented one, while grassroots innovation provides practical insights into understanding innovation from a local und bottom-up perspective.

Drawing from a comprehensive literature review on the integration of these topics, the article reflects on five dimensions: (a) concepts in use, (b) key fields and disciplines, (c) actors, (d) geographical areas, and (e) theoretical frameworks associated with grassroots innovation and social innovation. Despite the extensive research on both subjects in recent decades, there is surprisingly little literature specifically addressing grassroots innovation and embedding it within broader discourses on innovation and social innovation. Moreover, the analysis of similarities and relationships between the two concepts has been neglected. Therefore, this paper contributes to establishing a more comprehensive understanding of both concepts within the social science and innovation studies.

Accordingly, this literature review contributes to a better understanding of the concepts of social innovation and grassroots innovation in recent research. In particular, this review helps to identify their main differences, the contextualised use of the concepts and their use in different geographical areas. This paper also addresses the boundaries of grassroots innovation and social innovation as a major focus of research. The rationale for this review on social innovation and grassroots innovation comes from the review of different dimensions and concepts of social innovation (Maldonado-Mariscal, 2017: 39), including the role of organised civil society, collective power to innovate and promote social change at the local level. All these dimensions were contrasted with the idea and concept of grassroots innovation, where a gap in the literature was identified. While grassroots innovation can be located in social movement studies (Smith et al., 2016), there is still very little research on the links between social innovation and social movements (Maldonado-Mariscal, 2020). As a result, there is a lack of research explaining the differences and similarities between social innovations and grassroots innovations, as well as the complementarity between the two and their use in different contexts. Therefore, the following research question is presented here as the analytical basis for this review: *What are the differences and similarities presented by the literature between the concepts of social innovation and grassroots innovation?*

The following section presents definitions of both terms, which are recognised in the social innovation and innovation studies scientific communities. These definitions are presented for introductory purposes only.

## 2. Reviewing social innovation and grassroots innovation

Social innovations can be understood as new forms of organisation or new social institutions (Zapf, 1989), new social practices (Howaldt and Schwarz, 2010), new social relations, or the combination of different factors, processes or institutions to obtain better solutions (Maldonado-Mariscal, 2017). However, a narrower definition is needed to distinguish what is not a social innovation from what is. One of the most frequently used definitions of social innovation in the European context is that who define social innovations as “an intentional, targeted recombination or reconfiguration of social practices, which is attributable to certain actors or groups of actors in particular areas of action or social context, with the goal of solving problems or satisfying needs better than is possible based on established practices” (Howaldt and Schwarz, 2010: 54; Howaldt and Schwarz, 2021: 47).

Reviews of research on innovation and social innovation show a gap in the classification of social innovation and its types over the last 10 years (Rueede and Lurtz, 2012), while more recently there is more of a tendency towards specialisation of the field of social innovation in the literature (Maldonado-Mariscal and Alijew, 2023). These reviews reflect how some definitions remain fuzzy and an even more concrete definition is needed (Marques et al., 2018), since social innovation has an ambiguous aspect (Brandsen et al., 2016).

Grassroots innovation is defined by Seyfang and Smith (2007) as “networks of activists and organisations generating novel bottom–up solutions for sustainable development; solutions that respond to the local situation and the interests and values of the communities involved” (Seyfang and Smith, 2007: 585). Some authors using the definitions of grassroots innovation in transition studies differentiate between socio-technical innovations and social innovations. This last one is identified as a community-led (or social innovation) type of grassroots innovation (Seyfang and Smith, 2007; Seyfang and Haxeltine, 2012).

The definition of grassroots innovation focuses primarily on “novel” bottom-up solutions; similarly, social innovation recognises “new or recombined” social practices. Social innovation focuses more on new or recombined social practices than grassroots innovation. However, it has not yet been specified what kind of social practices we can delimit as social innovation. Grassroots innovation, on the other hand, refers to the creation of specific solutions based on the local situation, interests and values of the local group or community. The main difference between these concepts is that while grassroots innovation focuses on solutions, i.e., general solutions for local groups, social innovations refer to social practices to better meet needs. In terms of actors, social innovation refers to actors or groups of actors in specific contexts, while grassroots innovation recognises networks of activists and organisations. In this sense, it appears that the network element is more relevant in grassroots innovation concepts than in social innovation concepts. Based on these similarities, we investigate the differences and boundaries of these terms.

## 3. Method

This article provides an overview of grassroots innovation and social innovation concepts over the last 5 years, deconstructing these two concepts and some of their key dimensions. The method carried out for this review consists of a systematic literature review involving two concepts: “social innovation” and “grassroots innovation.” For this review, I conducted a search in Web of Science (WoS). This search was conducted for peer-reviewed articles from the last 5 years (2018–2022), including articles in English and Spanish. This search was conducted in the titles, abstracts, and keywords. The date for searching and downloading the articles was February 21, 2022. To avoid bias, all articles found with the search criteria were read and analysed, with a total of 51 articles published in WoS between 2018 and 2022.

A summary of the entire search is presented below. This search presents two rows of concepts, showing the focus of this review, in searches A) and B) presented in Table 1 here below. Where A) corresponds to the search for the combination of concepts social innovation and grassroots innovation and B) corresponds to the search for grassroots innovation. Initially, a search was also made for the concept of social innovation, where 1,457 articles were found, however, the reason for focusing only on A) and B) is that more research has been done on social innovation but less on the relationships between these two concepts “social innovation” and “grassroots innovation.” Therefore, this paper focused on searches A) and B). An overview of the papers can be find in Tables 2, 3 at the end of this article. This review is based on a total of 51 academic articles, of which 10 have the combination of grassroots and social innovation and 41 focus on grassroots innovation.

**Table 1:** Overview of the used criteria for the search and papers analysed.

	Concept 1	Concept 2	Period	Peer-reviewed article	Number of articles found	Number of articles analysed
A	Social innovation	Grassroots innovation	2018–2022	Yes	10	10
B	Grassroots innovation	–	2018–2022	Yes	41	41

**Table 2:** Overview of grassroots innovation and social innovation papers.

	Main concepts used in the theme grassroots innovation and social innovation	Scholars
Main concepts	Grassroots social innovation	Signori and Forno (2019)
	Grassroots, social innovation efforts	Banerjee and Shahan (2019)
	Social innovations, grassroots innovation enterprise	Yun et al. (2019)
	Grassroots social business	Colovic and Schruoffeneger (2021)
	Grassroots innovations	Duarte Alonso et al. (2020); Roysen and Mertens (2019); Jones et al. (2021); Molina-Betancur et al. (2021)
	Grassroots product-oriented social innovations	Zajda et al. (2020)
	Social entrepreneurship and grassroots innovation	van Lunenburg et al. (2020)

	Country	Scholars
Main geographical area	Italy	Signori and Forno (2019)
	India	Banerjee and Shahan (2019); Yun et al. (2019)
	Ghana	Yun et al. (2019)
	United Kingdom	Duarte Alonso et al. (2020)
	Brazil	Roysen and Mertens (2019); Colovic and Schruoffeneger (2021)
	Australia	Jones et al. (2021)
	Poland	Zajda et al. (2020)
	Netherlands	van Lunenburg et al. (2020)
	Colombia	Molina-Betancur et al. (2021)

	Theories and concepts	Scholars
Main theoretical frameworks	Collective consumption, sustainable behaviour, practices	Signori and Forno (2019)
	Solidarity	Banerjee and Shahan (2019)
	Community actions, system-changing innovation, open innovation policy	Yun et al. (2019)
	Human dimension, innovative practices, inclusive innovation, technology, new products, new skills	Duarte Alonso et al. (2020)
	Social practice theory, social value, institutional change	Roysen and Mertens (2019); Colovic and Schruoffeneger (2021)
	Index (Index of Implementation of Grassroots Product-Oriented Social Innovation)	Zajda et al. (2020)
	Scaling process (scaling up, scaling out)	van Lunenburg et al. (2020)
	Public spaces, agency, poverty, inequality, bottom-up solutions, collective	Molina-Betancur et al. (2021)
	Grassroots innovation, inclusive innovation	Jones et al. (2021)

**Table 3:** Overview of grassroots innovation papers.

	Main concepts used in the theme grassroots innovation	Scholars
Main concepts	Grassroots innovations (GI)	Boni et al. (2018); Jiménez-Martínez and García-Barrios (2020); Marletto and Sillig (2019); Boyer (2018); Lin et al. (2018); Pellicer-Sifres (2020); Belda-Miquel et al. (2020); Sillig (2022)
	Grassroots innovation (GRI)	Gupta (2020); Singh et al. (2018, 2020b); Ng et al. (2019); Nicolosi et al. (2018); Marletto and Sillig (2019)
	Grassroots movements	Hossain (2018)
	Grassroots investments, grassroots consumers	Tan et al. (2021)
	Local grassroots initiatives	Grandadam et al. (2021)
	Grassroots initiatives	Kump and Fikar (2021); Mehr et al. (2019)
	Grassroots-driven open communities (GOC)	Wolf and Bernhart (2022)
	Emergent grassroots innovations	Rasillo (2021)

	Country	Scholars
Main geographical area	Spain	Boni et al. (2018); Pellicer-Sifres (2020); Rasillo (2021); Belda-Miquel et al. (2020); Pellicer-Sifres et al., (2018)
	India	Sharma and Kumar (2018, 2019); Singh et al. (2018, 2020a,b, 2021); Sheikh and Bhaduri (2020); Patnaik and Bhowmick (2020); Hossain et al., (2021); Parwez and Chandra Shekar (2019); Sharma (2021); Wierenga (2020)
	Australia	Tan and Zuckermann (2021)
	Malaysia	Ng et al. (2019)
	Bangladesh	Khalil et al. (2020)
	USA	Nicolosi et al. (2018); Boyer (2018)
	China	Tan et al. (2021)
	Mexico	Jiménez-Martínez and García-Barrios (2020)
	England	Lang et al. (2020)
	Italy	Marletto and Sillig (2019); Seddone and Sandri (2020)
	Denmark, Netherlands, Sweden	Magnusson et al. (2021); Wolf and Bernhart (2022)
	Austria	Kump and Fikar (2021)
	Laos	Shin et al. (2019)
	Taiwan	Lin et al. (2018)
	Europe	Sillig (2022)

	Theories and concepts	Scholars
Main theoretical frameworks	Human development, multidimensional construct	Boni et al. (2018); Pellicer-Sifres (2020); Belda-Miquel et al. (2020)
	Tensions in grassroots movements	Hossain (2018)
	Socio-technical elements of GRIs, socio-technical transitions	Ng et al. (2019)
	Grassroots innovation for sustainability	Nicolosi et al. (2018); Jiménez-Martínez and García-Barrios (2020); Pellicer-Sifres et al., (2018)
	Innovation policy, circular economy	Ziegler (2019)
	Empowerment, inclusive innovation, transformative social learning	Tan et al. (2021)
	Innovation policies, community-based policies, structural change, regional level, local innovation dynamics	Grandadam et al. (2021)
	Strategic niche management, social capital theory	Lang et al. (2020)
	Multi-level framework	Belda-Miquel et al. (2020)
	Grassroots innovation pathways, urban mobility, empowerment	Marletto and Sillig (2019)
	Energy democratization	Magnusson et al. (2021)
	Systems thinking, feedback loops in scaling, paradox of diffusion	Kump and Fikar (2021)
	Diffusion: replication, up-scaling, translation, niche, dynamics of niche replication	Boyer (2018)
	New forms of activism, self-organised grassroots innovation	Rasillo (2021)
	Grassroots innovations processes, multi-level perspective, sociotechnical transitions, capability approach, multi-dimensional perspective	Belda-Miquel et al. (2020)
	Typology of innovation, civil society, sociological and ideological concerns, resistance, development framework, grassroots arena	Sillig (2022)

The details of the search were as follows:

- social innovation AND grassroots innovation, last 5 years, articles, peer-reviewed articles = 10 articles
- grassroots innovation NOT social innovation, last 5 years, articles, peer-reviewed articles = 41 articles

### 3.1. Rationale and limitations

The decision to first conduct a review of the combination of social innovation and grassroots innovation, as well as a review of the concept of grassroots innovation itself, was to identify possible similarities in the application of the concepts of grassroots innovation and social innovation. This review starts from the original idea of understanding what is the theoretical difference between the two concepts. The decision not to conduct a review of the concept of social innovation is due to the preponderance of the literature on social innovation. In this search were found 1,400 articles with social innovation according to the same criteria and the same time frame.

Some of the limitations of the review suggest the use of only one database, in this case WoS, as there may be different articles not visible in WoS, especially with literature from the global south. In addition, searching in only two languages is certainly a limitation. Nevertheless, this provides a good overview of main sources addressing both social innovation concepts and grassroots innovations.

## 4. Results

In this section, I present the analysis of the five dimensions of social innovation and grassroots innovation (a) concepts, (b) main fields and disciplines, (c) main actors involved, (d) geographical areas of research, and (e) theoretical approaches. This section also provides a contrast in terms of results. These results are presented first with the analysis of both social innovation and grassroots innovation, followed by the analysis of grassroots innovation.

### 4.1. Main concepts

#### 4.1.1. Social innovation and grassroots innovation

Ten scientific papers were found with the combination of concepts of social innovation and grassroots innovations (section methodology, section A). The most commonly used terms in the search for social innovation and grassroots innovation were: grassroots innovation (Roysen and Mertens, 2019; Duarte Alonso et al., 2020; Jones et al., 2021; Molina-Betancur et al., 2021), grassroots social innovation (Banerjee and Shahan, 2019; Signori and Forno, 2019), grassroots social business (Colovic and Schruoffeneger, 2021), and finally, social entrepreneurship and grassroots innovation (van Lunenburg et al., 2020). A graphical representation of this category is presented in Table 2.

The concept grassroots social innovation was used in two papers. First, Signori and Forno (2019) introduce here the concept of grassroots social innovation niches, referring to both social innovation and grassroots innovation approaches. In terms of social innovation, they base their understanding on the fact that social innovations are related to at least two approaches: innovations as a response to market and state inefficiencies (Signori and Forno, 2019: 804), and, as socio-political transformations (Moulaert, 2009: 12). As for grassroots innovation, Signori and Forno (2019) refer to community-led grassroots innovation with the same definition as presented in the introduction of this paper (Seyfang and Smith, 2007). Although this paper combines the two main concepts of this review, and provides an introduction to both concepts and frameworks, it does not offer any specific definition for grassroots social innovation niches introduced in the paper. Second, Banerjee and Shahan (2019) also made use of the concept grassroots social innovation, however, no definition of this concept was offered. In this paper, the theoretical framework of community-entrepreneurship was used, introducing the “community-preneurship” term. Banerjee and Shahan (2019: 127) used the term for the context of the global south. These scholars combine the two perspectives of social innovation (new business practices) and grassroots innovation (community innovation), but offer a contested concept applied to the global south, which has a contextualised aspect.

#### 4.1.2. Grassroots innovations

Forty-one scientific papers were found with the concept of grassroots innovations (see methodology section B). The term most commonly used in the papers was grassroots innovations, abbreviated as (GI) or (GRI) (Boni et al., 2018; Boyer, 2018; Hossain, 2018; Lin et al., 2018; Nicolosi et al., 2018; Singh et al., 2018; Marletto and Sillig, 2019; Ng et al., 2019; Belda-Miquel et al., 2020; Gupta, 2020; Jiménez-Martínez and García-Barrios, 2020; Pellicer-Sifres, 2020; Singh et al., 2020b; Sillig, 2022). Other terms used in this search were grassroots movements (Hossain, 2018), grassroots initiatives (Grandadam et al., 2021; Kump and Fikar, 2021; Dana et al., 2021; Mehr et al., 2019), grassroots NGOs (Farid, 2019); or emergent grassroots innovations (Rasillo, 2021). A summary of this category is presented in Table 3.

When analysing the concepts of grassroots innovation, it was found that this concept was used differently in English than in Spanish in one article (Boni et al., 2018). For example, the abstract in English makes use of the concept of grassroots innovations, while throughout the literature and title of the article, it refers to collective social innovation (innovación social colectiva). This paper defines this concept as “collective social innovation (CSI) that explicitly seeks the transformation of socio-technical regimes” (Boni et al., 2018: 67, own translation), and is not limited to grassroots innovations.

On the other hand, the concept of grassroots movements, was used in only one article (Hossain, 2018). In this paper, the author defined it as “GMs are movements that emerge from the local level with a bottom-up approach and diffuse throughout the state and at the national level” (Hossain, 2018: 63). That paper is based on a systematic literature review of articles on grassroots innovations, and includes both concept of grassroots innovation and grassroots movements as an umbrella to identify movements, collectives, and informal community groups. The definition used in that article for grassroots innovation is the same mentioned in the introduction of this paper, as the one used in Seyfang and Smith (2007). Other works use the same definition of grassroots innovation but establish a stronger link between grassroots innovation and the socio-technical transitions approach (Pellicer-Sifres, 2020). They underline the characteristics of the origin of grassroots innovation, for example: grassroots innovation are originated by civil society, they are based on social experiments using innovative technologies, values or institutions (Jiménez-Martínez and García-Barrios, 2020).

### 4.2. Main fields and disciplines

#### 4.2.1. Social innovation and grassroots innovation

The combination of both concepts social innovation and grassroots innovations is mostly represented in the disciplines of Business, Management, and Sociology. In this literature review, three main fields were identified: sustainability, social business, and informal innovation. Firstly, within the sustainability field, the main topic was new lifestyles, which is related to food consumption, sustainable development and consumption, water management, but also to sustainable practices, such as ecovillages, and urban regeneration. Secondly, social business, under this topic was mainly found business models, open innovation, and community entrepreneurship. Finally, a third field, but less addressed, was the topic of artistic training, informal grassroots training and traditional indigenous art.

#### 4.2.2. Grassroots innovations

The concept of grassroots innovations is mostly represented in five categories or disciplines, such as Environmental Issues, Business and Management, Development Studies, Regional Urban Planning, and Economics. More concretely, the main themes addressed in the papers analysed focused on sustainability, grassroots in the informal sector, networking capacities and grassroots innovations in ICT. Interestingly, most of the papers focused in the topic of sustainability, for example in sustainable practices (Boyer, 2018; Hossain, 2018; Nicolosi et al., 2018; Jiménez-Martínez and García-Barrios, 2020; Singh et al., 2020b), energy transformation, energy transitions or energy democratization (Boni et al., 2018; Pellicer-Sifres, 2020; Magnusson et al., 2021), sustainable transformation and sustainable transitions (Lin et al., 2018; Marletto and Sillig, 2019; Shin et al., 2019; Lang et al., 2020; Kump and Fikar, 2021), or even climate change adaptation (Khalil et al., 2020). More specific fields refer to water innovation (Ziegler, 2019) and waste management (Jiménez-Martínez and García-Barrios, 2020).

Less papers focused on how grassroots innovations emerged in the informal sector (Sharma and Kumar, 2018, 2019; Sheikh and Bhaduri, 2020, 2021), and learning practices and networking capacities (Singh et al., 2020b, 2021). Other themes identified were ICT supporting the development of grassroots innovation (Singh et al., 2018; Shin et al., 2019), grassroots innovation in language (Tan and Zuckermann, 2021), and rural grassroots innovations (Ng et al., 2019), these themes were found seldom.

**Figure 1:**
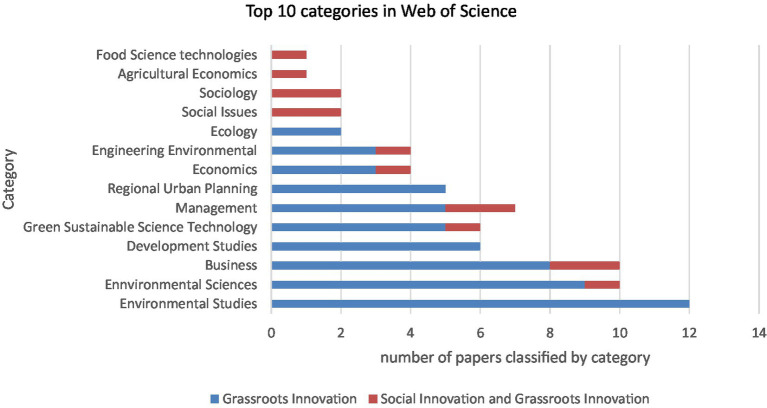
Comparison of the Web of Science (WoS) categories of all analysed articles. Self-elaborated based on WoS, database from February 21, 2022.

By comparing the disciplines in all analysed articles based on the WoS classification, and including both searches (A and B), we can recognise the following two aspects. One, the combination of the concepts of social innovation and grassroots innovations (A) is mainly anchored in Business, Management and Sociology or Social Issues (see red bars in Figure 1). Two, the grassroots innovations concept (presented as B) is significantly represented in at least five categories (see blue bars in Figure 1): Environmental Issues (such as Environmental Sciences, Green Sustainable Sciences, Environmental Engineering and Ecology), Business and Management, Development Studies, Regional Urban Planning, and Economics. This classification shows a significant difference in the focus of articles on social innovation and grassroots innovations published in WoS in the last 5 years (See Figure 1).

### 4.3. Main actors

#### 4.3.1. Social innovation and grassroots innovation

When analysing the dimension of actors considered in the papers included in this review, a diversity of actors was found by the combination of grassroots innovation and social innovation. The main actors identified are, for example, community entrepreneurship and social enterprises (Banerjee and Shahan, 2019), grassroots organisations (Zajda et al., 2020; Jones et al., 2021), NGOs, arts centre managers, consumers, but also the community actors for the appropriation of public space (Molina-Betancur et al., 2021).

#### 4.3.2. Grassroots innovations

Similar to the combination of both concepts, a diversity of actors was found. The main actors identified are, for example, community (e.g., community-led housing, grassroots communities), organised civil society, cooperatives (Belda-Miquel et al., 2020), or networks of cooperatives (e.g., ecovillages) (Rasillo, 2021). But also NGOs and micro-enterprises (Lin et al., 2018), and project leaders, women from NGOs and indigenous communities (Tan and Zuckermann, 2021).

### 4.4. Geographical area

#### 4.4.1. Social innovation and grassroots innovation

This dimension of geographical area refers to the location in which the papers focused their research. Tables 2, 3 provide a graphical representation of this dimension. In cases where no geographical area was indicated, the institutional affiliation of the first author was taken into account. In the analysis of the geographical area of the two concepts, two countries were identified as central: India and Brazil. At least two papers were geographically focused on India (Banerjee and Shahan, 2019; Yun et al., 2019) and Brazil, respectively (Roysen and Mertens, 2019; Colovic and Schruoffeneger, 2021). Other geographical areas mentioned were Italy, Ghana, United Kingdom, Australia, Poland, Netherlands, and Colombia. Most of the articles in this review focus on Europe (4 articles) and South America (3 articles), and finally Asia and Africa (1 article) respectively. As a comparative overview, most papers are focused on countries of the global South, although the role of Europe remains important.

#### 4.4.2. Grassroots innovations

In a clustering analysis of the documents on grassroots innovation, two countries were found to be in the spotlight: India (Sharma and Kumar, 2018, 2019; Singh et al., 2018; Patnaik and Bhowmick, 2020; Sheikh and Bhaduri, 2020; Singh et al., 2020a,b, 2021; Hossain et al., 2021; Parwez and Chandra Shekar, 2019; Sharma, 2021; Wierenga, 2020) and Spain (Boni et al., 2018; Belda-Miquel et al., 2020; Pellicer-Sifres, 2020; Rasillo, 2021; Pellicer-Sifres et al., 2018). Other regions mentioned in the grassroots innovation papers were Australia, Malaysia, Bangladesh, United States, China, Mexico, England, Italy, Denmark, Netherlands, Sweden, Austria, Laos, and Taiwan. In contrast to social innovation and grassroots innovation, most of the articles in this category focused on Asia (13) and Europe (12) and to a much lesser extent on North America and Oceania, while no articles focused on Africa.

### 4.5. Theoretical frameworks

#### 4.5.1. Social innovation and grassroots innovation

The dimension of theoretical frameworks included in the reviewed papers represent one of the most varied and interesting dimensions. The papers reflect a very broad approach to studying grassroots innovations and social innovations (See Table 2). A common framework in use was collective, solidarity, community actions, as well as the use of agency and bottom-up solutions (Banerjee and Shahan, 2019; Signori and Forno, 2019; Molina-Betancur et al., 2021). Theories highlighted here were sustainable practices and social practice theory (Roysen and Mertens, 2019; Signori and Forno, 2019). In addition, the framework of inclusive innovation with the combination of technology (Duarte Alonso et al., 2020), and innovation with poverty and inequality framework were mentioned in one paper, respectively (Molina-Betancur et al., 2021). Finally, the use of system-changing innovation framework, open innovation and scaling process (up and out) as a common part of the innovation process had great relevance (Yun et al., 2019; van Lunenburg et al., 2020).

#### 4.5.2. Grassroots innovations

In the study of grassroots innovations, at least five main theoretical frameworks were identified: (a) capabilities approach, (b) multilevel perspective, (c) empowerment, (d) socio-technical transitions and, finally, (e) resistance and tension in grassroots innovation (see Table 3). First, one of the most common frameworks used were concepts of human development, multilevel perspective and capabilities approach (Boni et al., 2018; Belda-Miquel et al., 2020; Pellicer-Sifres, 2020). Secondly, the framework of empowerment, such as new forms of activism, civil society and inclusive innovation (Marletto and Sillig, 2019; Rasillo, 2021; Tan et al., 2021; Sillig, 2022). Thirdly, a frequently used framework was that of socio-technical transitions (Ng et al., 2019; Belda-Miquel et al., 2020; Pellicer-Sifres, 2020). Finally, resistance and tension within grassroots movements were identified (Hossain, 2018; Sillig, 2022). Other approaches less used were innovation policy and community-based policies (Ziegler, 2019; Grandadam et al., 2021), as well as the concepts of scalability and diffusion (Boyer, 2018; Kump and Fikar, 2021) or innovation typologies (Tan and Zuckermann, 2021; Sillig, 2022).

## 5. Discussion

This review shows there is limited literature that specifically addresses grassroots innovation and its place within broader discourses of social innovation. Despite some similarities when looking at case studies or local initiatives (Grandadam et al., 2021), the literature on grassroots innovation is more anchored in concepts of empowerment, social movements, new forms of activism and, specifically, socio-technical transitions. As far as can be observed, the scarce literature on grassroots innovations and social innovation shows a gap in the dialogue between the two disciplines.

The main differences in the literature on social innovation and grassroots innovation show that when the combination of the concepts social innovation + grassroots innovation is used, it is often understood as grassroots social enterprises or entrepreneurial initiatives. However, when grassroots innovation was used as a stand-alone concept, it was mostly understood in the context of bottom-up movements in societies. The results show that when the concept of social innovation was added, an element of ambiguity had been added as well, as it was sometimes understood as social enterprises and sometimes referred to local collective processes of transformation. In terms of theories, social practice theory, for example, was mostly used for social innovation frameworks, but not for grassroots innovation. We observed that the boundaries of the concept of grassroots innovation seem to be better delimited than those of social innovation, since a greater consensus was found in the definition of grassroots innovation than in that of social innovation. The classification of fields shows a significant difference in the focus of articles on social innovation and grassroots innovations published in the WoS over the last 5 years (see Figure 1). This means that there is a greater focus of the concept grassroots innovations on environmental issues, while the term social innovation is used more generalist and within sociological frameworks.

The similarities between grassroots innovations and social innovation frameworks were not clearly found in the literature. The best examples of this are two papers that combine both concepts (Banerjee and Shahan, 2019; Signori and Forno, 2019). The first paper introduces and defines both approaches, however, it does not offer any specific definition, i.e., grassroots social innovation. The second paper, also makes use of the concept of grassroots social innovation, but the interpretation focuses on community entrepreneurship, creating a new concept of “community-preneurship,” which tries to combine both theories applied to the context of the global south, but with a specific focus on entrepreneurship. However, there seems to be a high consensus in the use of the definition of grassroots innovation, as many authors use the same definition of grassroots innovation as Seyfang and Smith (2007).

Although we found more differences than similarities in the use of the concepts of social innovation and grassroots innovation, there is a great potential for complementarity of these concepts. This potential is shown especially in the deconstruction of the different dimensions of the concepts, using different theories and examples. But also in the discussion of the different social, institutional and political dimensions of the innovations. In this sense, the contribution of grassroots innovations with a strong focus on socio-technical transitions and sustainability issues can contribute to specific discussions and concrete examples on social innovation, and the underlying tension of innovation. Similarly, social innovation can further contribute to the debate on grassroots innovations by using and understanding not only the agency of actors, but also the innovation ecosystem, actors and types of innovation.

Having observed trends in the use of both concepts in different geographical regions, I strongly suggest that both concepts should be studied by contrasting them. A possible hypothesis suggests that there are considerable differences in the use of both concepts in the Global North and the Global South. Our results show that the combination of concepts (social innovation and grassroots innovations) is very common in the global south, for example, in countries such as India and Brazil, (Banerjee and Shahan, 2019; Roysen and Mertens, 2019; Yun et al., 2019; Colovic and Schruoffeneger, 2021) while the use of grassroots innovations as stand-alone concepts is much broader and strongly used in India (Sharma and Kumar, 2018, 2019; Singh et al., 2018, 2020a,b, 2021; Patnaik and Bhowmick, 2020; Sheikh and Bhaduri, 2020). However, in order to test this hypothesis a lager review should be conducted, including papers in other languages such as Portuguese and French to get a variation of more research from the Global South. I therefore encourage further research with this possible research questions: Are there considerable differences in the use of the two concepts in the Global North and the Global South? What cases and types of grassroots innovation are described in the different regions? To what extent are grassroots innovations social innovations? Are there strong links between social innovations, social movements and grassroots innovation, and in which regions is this link strongest?

In summary, this review explored the concepts of social innovation and grassroots innovation through an analysis of relevant scientific articles. The combination of social innovation and grassroots innovation resulted in 10 articles, where the most frequently used terms were grassroots innovation, grassroots social innovation, grassroots social enterprise and social entrepreneurship and grassroots innovation. In contrast, the review on the term grassroots innovations revealed a stronger consensus on the definition, with several studies emphasising its link to socio-technical transitions or highlighting characteristics such as origin in civil society and the use of innovative technologies, values or institutions. The concept of grassroots innovations differs slightly between English and Spanish, as exemplified by the use of collective social innovation in one article in the Spanish literature. The results contribute to a better understanding of grassroots and social innovation, showing its key dimensions and providing a basis for future research in this field.

The analysis of actors in the review of social innovation and grassroots innovation revealed a wide range of diverse actors involved in these fields. In the case of social innovation actors involved include community entrepreneurs, social enterprises, grassroots organisations, NGOs, arts centre managers, consumers, as well as the community itself. In the literature of grassroots innovation, this dimension included the community, civil society, but specified as cooperatives, cooperative networks such as ecovillages, NGOs, micro-enterprises and local communities. This diversity of actors underlines the multifaceted nature of grassroots and social innovation, and highlights the importance of inclusive and participatory approaches to fostering transformative change at different levels of society.

In terms of geographical areas, the combination of social innovation and grassroots innovation revealed the prominence of particular countries, such as India and Brazil. Most of the work on social innovation focused on Europe, South America and, to a lesser extent, Asia and Africa, highlighting the global representation with a significant focus on the global South. Similarly, in the context of grassroots innovation, India and Spain were identified as countries with a strong research emphasis. Several papers studied the landscape of grassroots innovation in India, while Spain also made notable contributions with this regional focus. Overall, the geographical dimension of social and grassroots innovation research reflects a diverse global landscape, with a wide range of countries and regions contributing to the understanding of innovation dynamics within their specific contexts. This underlines the need for context-specific approach when studying and applying social and grassroots innovation practices around the world.

Finally, the theoretical frameworks employed in the papers on social innovation and grassroots innovation demonstrate a very big diversification for understanding these concepts. Common frameworks emerged, including the use of collective, solidarity, community actions, agency, and bottom-up solutions. Sustainable practices and social practice theory were also common frameworks, along with inclusive innovation and frameworks addressing poverty and inequality. Additionally, system change innovation, open innovation, and scaling processes were identified as integral parts of the innovation process. More specifically in the context of grassroots innovations, also a diversity of theoretical frameworks was observed. The concept of human development, the multilevel perspective, and the capabilities approach were commonly employed. In this papers the dimension of empowerment, new forms of activism, and inclusive innovation served as important frameworks. Socio-technical transitions and the analysis of resistance and tension within grassroots movements were frequently used approaches, whereas less commonly utilized frameworks included innovation policy, community-based policies, scale and diffusion concepts, or innovation typologies.

## 6. Conclusion

In conclusion, while social innovations and grassroots innovation focused on the disciplines of Business, Management and Sociology, grassroots innovation studies had a strong tendency towards Environmental Issues, but also Development Studies and Regional Development.

In terms of actors, studies on social innovation and grassroots innovation focused on social entrepreneurs in social enterprises, while studies on grassroots innovation focused more on community-led initiatives, civil society in the form of organised society, cooperatives and local leaders.

In terms of geographic area, India plays an important role in both analyses, as they are the main regional foci of the grassroots and social innovation papers. First, the analysis of social innovation and grassroots innovations (A), India was the most important focus, whereas Brazil the second most important regional focus. Second, the analysis of grassroots innovations (B), similarly, the main regional focus was India and secondly Spain. Overall, most of the social and grassroots innovation papers focused on Europe and South America, while the grassroots innovation papers mainly focused on Asia and Europe.

One of the most interesting aspects of the results found in this revision are the variety of theoretical concepts extracted from both concepts. While social innovation and grassroots innovation had a more sociological approach, with theories on social practice and concepts linked to collective, solidarity and community actions, the theoretical frameworks of the grassroots innovation focused on power relations and socio-technical transition.

### 6.1. Outlook

In the analysis of the combination of the concepts social innovation and grassroots innovation, there is a relevant focus on the countries of the global South, although the role of Europe remains highly representative. This review shows the need for further research on grassroots innovation and social innovations. Some results show that grassroots innovation is a concept preferentially used in sustainable practices. However, the term grassroots innovation seems to be mainly focused in the global North in topics such as sustainable practices, energy transformation and sustainable development, while in the global South it is often used in the informal sector and in the business context. To further test this hypothesis, a broader review would be necessary in order to have the same amount of articles from the global south and the global north and to collect evidence from these. Therefore, I encourage scholars to collaborate in future research for a better understanding of these two concepts, social innovation and grassroots innovation. Such research is crucial to advance the understanding of new and innovative practices of local and community-based initiatives. In addition, both concepts and an overview of the review of these concepts in innovation debates, further research is needed to explore the boundaries of social innovations, grassroots innovations and social movements. Two central questions for future research are the following: To what extent are social innovations a form of grassroots innovation, and what dimensions of social innovations and grassroots innovations build a social movement?

## Author contributions

KM-M developed the entire concept, methods and carried out the analysis of this article. She wrote the entire article.
